# Fault Diagnosis Method of Rolling Bearing Based on 1D Multi-Channel Improved Convolutional Neural Network in Noisy Environment

**DOI:** 10.3390/s25072286

**Published:** 2025-04-04

**Authors:** Huijuan Guo, Dongzhi Ping, Lijun Wang, Weijie Zhang, Junfeng Wu, Xiao Ma, Qiang Xu, Zhongyu Lu

**Affiliations:** 1 Department of Engineering, Huanghe Science and Technology University, Zhengzhou 450045, China; guohuijuan0320@126.com (H.G.); wujunfeng301@163.com (J.W.); 2 School of Mechanical Engineering, North China University of Water Resources and Electric Power, Zhengzhou 450045, China; 201721704@stu.ncwu.edu.cn (D.P.); zhangweijie905@163.com (W.Z.); maxiao@ncwu.edu.cn (X.M.); 3 School of Computing and Engineering, University of Huddersfield, West Yorkshire HD1 3DH, UK; q.xu2@hud.ac.uk; 4 The Glass Box, 6 Friendly Street, Huddersfield HD1 1RD, UK

**Keywords:** deep learning, fault diagnosis, Convolutional Neural Networks, gearbox, vibration signal

## Abstract

The vibration signal of mechanical equipment in operating environments is the key to describing fault characteristics, but due to thez influence of equipment density and environmental interference, the accuracy of fault diagnosis is often affected by noise. In this paper, a fault diagnosis method based on a 1D Multi-Channel Improved Convolutional Neural Network (1DMCICNN) is proposed. By introducing BiLSTM, an attention mechanism and a local sparse structure of a two-channel Convolutional Neural Network, the feature information of the noisy timing signal is fully extracted at different scales while reducing the computational parameters. The model is verified through experiments under different signal-to-noise ratios and loads. The results show that the accuracy of 1DMCICNN is 98.67%, 99.71%, 99.04%, and 99.71% on different load and speed datasets. Meanwhile, compared with the unoptimized two-channel Convolutional Neural Network, the training parameters are reduced by 55.58%.

## 1. Introduction

### 1.1. Related Work

With the rapid development of science and technology, intelligent, efficient, and automated machinery and equipment have been widely used in heavy-duty tasks in every corner of the equipment manufacturing industry [1]. A rich variety of rotating machinery plays an extremely important role in the process of industrial development, including aerospace, rail transportation, military equipment, nuclear power, wind power, etc. [2,3].

The working environment and working conditions of rotating machinery are complex and changeable. For example, they could be under high-load and high-speed conditions for a long time [4,5]. The characteristics of the working environment of rotating machinery may significantly increase the difficulty in repairing machinery and equipment, as well as the cost of maintenance. The machinery and equipment need to be repaired and maintained in a timely and effective way in the event of failure or impending failure; otherwise, this will not only affect the normal operation of the equipment but also cause economic losses and serious casualties in enterprises [6,7,8]. The rolling bearing is the core component of rotating machinery, known as the “industrial joint”, which is one of the most prone to failure parts in the process of industrial production equipment operation. The rolling bearing is easy to assemble and disassemble and easy to lubricate, and its structure can transform sliding friction into rolling friction to reduce wear [9,10,11,12].

Improving the diagnosis and monitoring ability of bearing fault types is beneficial to the service life and increases the safety of machinery and equipment. The causes of bearing fault signals generally include temperature, oil samples, vibration, sound, etc. The vibration signal of a fault bearing contains further information, such as the load, speed, working noise, etc. The bearing fault diagnosis method for the vibration signal has become one of the main research directions in the topic area [13,14].

The development of fault diagnosis methods for rolling bearings has gone through three different stages: (1) use of the traditional method of signal analysis; (2) extracting fault features using analysis methods for collected signals in the time domain, the frequency domain, and the time–frequency domain; and (3) using the deep learning algorithm deep neural network (DNN) model to automatically extract the deep features of the fault data, accurately identify the fault types, and reduce dependence on expert experience. The DNN model can make full use of massive data and the current hardware level [15].

In the field of fault diagnosis, the running state of bearings can be assessed according to the time domain features that are obtained after signal processing [16]. However, in the actual diagnosis process, it is difficult to apply the same feature in the discrimination of various faults; thus, it is necessary to determine which time domain features will be chosen according to the actual situation [17]. In the time domain, features like the root mean square, the average amplitude, the peak value, and kurtosis are generally included.

However, in practical applications, the time domain analysis method cannot determine the specific characteristics of the fault, and diagnosis accuracy is low [18], so it is less applicable. The fault diagnosis method using frequency domain analysis is used to convert the time domain signal to the frequency domain signal through signal processing, and the most common method used to convert the time domain to the frequency domain is Fourier Transform (FT). After time domain conversion, the fault frequency, harmonic frequency, and other information of the fault bearing are obtained, and then the frequency domain dimension index is used to diagnose the bearing fault. Commonly used methods include power spectrum analysis, cepstrum analysis, and envelope spectrum analysis [19].

Consideration of the mechanical equipment used in an operating environment is very complex because the collected signals are often nonlinear and non-stationary [20]. The method of bearing fault diagnosis based on frequency domain analysis could achieve good results when the collected signals are relatively stationary, but it is difficult to process non-stationary signals [21]. The time–frequency domain analysis method, which is advantageous for processing non-stationary signals, can simultaneously establish the two-dimensional relationship between time and frequency. Common time–frequency analysis methods include Short-Time Fourier Transform (STFT) [22], Wavelet Transform (WT) [23], and Empirical Mode Decomposition (EMD) [24].

Although the method of time–frequency analysis has solved some problems of feature extraction in nonlinear and non-stationary signals compared with the method of frequency domain analysis, both methods have their own limitations. For example, in the method of Empirical Mode Decomposition, the mode aliasing phenomenon will occur in the intrinsic mode function (IMF) component decomposition process, and the features of different scales will appear in the same IMF component [25]. Similar problems also appear in the method of Wavelet Transform [26]. Although Wavelet Transform can analyze the signal locally, it is difficult to determine the appropriate function of the wavelet basis, resulting in poor generalization performance of the Wavelet Transform. Thus, it is difficult to express the fault characteristics.

With the continuous improvement and development of AI technologies, the use of machine learning for fault diagnosis has advantages in fault pattern recognition, but it is difficult to effectively extract the features in the data. Therefore, it is often combined with other methods that perform well in feature extraction [27]. The most commonly used machine learning methods are the Artificial Neural Network (ANN) [28], the Extreme Learning Machine (ELM) [29], the Support Vector Machine (SVM) [30], and K-Nearest Neighbor (KNN) [31].

Deng et al. [32] used the Particle Swarm Optimization (PSO) algorithm to optimize the parameters of the least squares Support Vector Machine (LS-SVM) and improved the classification accuracy. Surajkumar et al. [33] combined Dimensional Analysis (DA) with an Artificial Neural Network and used the actual error and error performance to evaluate the model’s performance. Meanwhile, they tested an experimental platform and proved the good performance of the proposed method. Lu et al. [34] proposed a case-based reconstruction algorithm to adaptively locate the nearest neighbor of each test sample, which can simultaneously use parameters for the classification of bearing faults. Prasad et al. [35] extracted the basic bearing frequency from the vibration response and selected extracted values as a feature to input into the K-Nearest Neighbor network for fault diagnosis. They achieved 98.5% recognition accuracy in experiments under different working conditions.

Machine learning has achieved good performance in the field of fault diagnosis, but traditional machine learning is limited by small data sample sizes and computing units, so it is difficult to express the complexity of fault diagnosis, as the architecture of machine learning is insufficient to achieve a good diagnosis result, especially for the feature extraction of a large number of high-order data [36].

The deep learning method has achieved better performance than machine learning in bearing fault diagnosis because of its adaptive characteristics in processing big data and the deep mechanism of the nonlinear computing unit [37]. In recent years, research on deep learning has attracted the attention of more and more scholars. It is more widely used in object recognition, image segmentation, speech recognition, machine health detection, and medical health, as well as engineering and technologies [38,39,40,41]. The commonly used deep learning methods are Convolutional Neural Networks (CNNS) [42], the Deep Belief Network (DBN) [43], and the Generative Adversarial Network (GAN) [44], etc.

CNN is a deep feedforward neural network that can adaptively extract the basic features in the collected signals so as to complete accurate feature selections. In the early stage, the two-dimensional structure of CNN was used in the field of fault diagnosis, and the one-dimensional data were converted into two-dimensional data for processing. Cheng et al. [45] proposed a fault diagnosis method combining the Convolutional Neural Network and the Extreme Learning Machine. Firstly, the original vibration signal was processed through continuous Wavelet Transform, and then high-level features were extracted using CNN and ELM was used as a classifier to improve classification performance. Gong et al. [46] proposed an improved Convolutional Neural Network Support Vector Machine, which can directly input the original data from multiple sensors into the CNN–Softmax model, which inputs the extracted feature vector into the Support Vector Machine for fault classification. The results are better than those obtained through other methods, such as the SVM and K-Nearest Neighbor. Jiang et al. [47] considered the multi-scale characteristics of the gearbox vibration signal and effectively learned deep fault features through the hierarchical learning structure of the convolutional layer and the pooling layer, which improved the feature learning ability.

The Deep Belief Network is composed of a multi-layer restricted Boltzmann machine and a BP neural network [48]. The training process of the network model can be divided into two parts, pre-training and reverse fine-tuning, which have the advantages of low computational cost and easily fall into local optimum when initializing weight parameters [49]. Deng et al. [50] proposed an improved Multi-Swarm Intelligence Quantum Differential Evolution (MSIQDE) algorithm to optimize DBN parameters, which avoids premature convergence and improves the global search ability. Experimental results show that MSIQDE–DBN can obtain greater classification accuracy than other compared methods.

The Generative Adversarial Network (GAN) consists of two parts: the generator function and the discriminator function. The GAN has been applied to the field of fault diagnosis by more and more scholars because of its excellent generation ability. Liu et al. [51] proposed a fault diagnosis method combining an improved multi-scale residual Generative Adversarial Network and a feature enhancement driven capsule network, which improved the performance of imbalanced fault pattern classification in practical engineering contexts. Zhou et al. [52] proposed a new generator and discriminator of a Generative Adversarial Network (GAN). The generator was improved by extracting fault features through Autoencoder (AE). Experimental results show the effectiveness of the proposed method. Mao et al. [53] used the spectrum data generated after processing the original data as the input for the GAN. The GAN generated synthetic samples of a few fault categories according to the real samples and then put the synthetic samples into the training set. The experiments proved the improvement of the generalization ability of the model.

In the actual engineering context, the fault position of the bearing will cause unsteady vibration when it is in contact. The characteristic frequency of the bearing fault is related to the change of the rotational speed of the mechanical equipment shafting [54,55,56]. Under different conditions, such as different loads, the fault signals are varied. Li et al. [57] proposed a fault diagnosis method based on deep learning and knowledge graph fusion. They used bearing data knowledge to extract entities and a multi-scale optimization Convolutional Neural Network to classify fault types. Experimental results show that it has good performance in various operating conditions and noise environments. In order to solve the problem of low accuracy of bearing fault diagnosis under unknown working conditions, Kong et al. [58] added a weight allocation mechanism to the feature extraction layer in the Convolutional Neural Network and used the dataset of Case Western Reserve University (CWRU) for the experiments. The results show that the bearing fault recognition ability is effectively improved.

### 1.2. Document Structure

This paper is mainly divided into three parts. The first part introduces the design and optimization of the 1DMCICNN, expounds the principle and role of the module introduced in the model, and selects the optimizer with better performance through experimental comparison. Section 2 describes the structure, parameters, and diagnosis process of the 1DMCICNN. Thirdly, the source of experimental data and the experimental design are introduced, the comparison experiments of variable working conditions in a noisy environment are carried out, and the experimental results are analyzed from different angles.

In this paper, a rolling bearing fault diagnosis model based on the One-Dimensional Multi-Channel Improved Convolutional Neural Network (1DMCICNN) in a noisy environment is constructed. Firstly, the basic principles of the Bi-Directional Long Short-Term Memory (BiLSTM) Network, the local sparse structure, the improved optimizer, and the improved activation function are introduced. The attention mechanism, the BiLSTM, and the local sparse structure are introduced into the Improved Multi-Channel Convolutional Neural Network and integrated into an adaptive anti-noise bearing fault diagnosis model for noisy environments. The improved performance of the proposed model is proved by experiments under single working condition, various working conditions, multiple load conditions under different signal-to-noise ratio conditions, and visual analysis of the results. Finally, the outcome improves the interpretability of the model.

## 2. Design and Optimization of 1DMCICNN

### 2.1. Bi-Directional Long Short-Term Memory Network

**Figure 1:**
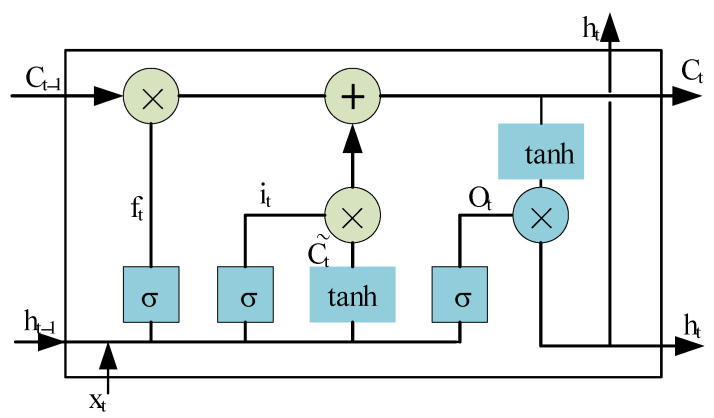
Structure diagram of LSTM.

The Long Short-Term Memory Network (LSTM) was developed from the recurrent neural network (RNN) by Hochreiterand Schmidhuber in 1998. After training, the RNN has good performance for sequence data, but problems of gradient disappearing and gradient explosion will occur in the training process. The possibility of gradient explosion and gradient disappearance can be effectively reduced by introducing LSTM with a unique gating mechanism. The structure of LSTM is shown in Figure 1.

LSTM can learn long-term dependencies between two sequences because it has long-term memory. The key to LSTM is to pass information from cell state $c_{t-1}$. to cell state $c_{t}$ and selectively retain desired characteristics in the process. The LSTM unit consists of input gate $i_{t}$, forgetting gate $f_{t}$, and output gate $o_{t}$. The function of the input gate is to determine the retention degree of the input at t time, the function of the forgetting gate $f_{t}$ is to selectively abandon the information in some memory units, and the output gate $i_{t}$ determines the output at t time through the cell state $c_{t}$. Under the joint action of the three gate mechanisms, the LSTM unit can realize the selection of information, the flow of information, and the preservation of long-term information in the data. The mathematical expressions of the three gates are as follows:

(1) $$i_{t}=\sigma \left(w_{i}\left[h_{t-1},x_{t}\right]+b_{i}\right)$$

(2) $$f_{t}=\sigma \left(w_{f}\left[h_{t-1},x_{t}\right]+b_{f}\right)$$

(3) $$o_{t}=\sigma \left(w_{o}\left[h_{t-1},x_{t}\right]+b_{o}\right)$$

The mathematical formula for calculating the temporary cell state ${\tilde{c}}_{t}$, the current cell state $c_{t}$, and the hidden layer state $h_{t}$ at the current moment are as follows:

(4) $${\tilde{c}}_{t}=\tan h\left(w_{c}\left[h_{t-1},x_{t}+b_{c}\right]\right)$$

(5) $$c_{t}=f_{t}\circ c_{t-1}+i_{t}\circ \tan h\left(w_{c}\left[h_{t-1},x_{t}+b_{c}\right]\right)$$

(6) $$h_{t}=o_{t}\circ \tan h\left(c_{t}\right)$$

From Formula (1) to Formula (6), σ is the activation function Sigmoid, ∘ is the element-by-element product operation, $w_{x}$ is the weight of the corresponding gate, $h_{t-1}$ is the hidden layer state at t−1, $x_{t}$ is the input at t, $b_{x}$ is the bias of the corresponding gate, [ ] is the series between vectors, $c_{t-1}$ is the cell state at t−1, $o_{t}$ is the output gate value at t, and $f_{t}$ is the forgotten gate value at t. $i_{t}$ is the value of the t-moment memory gate.

**Figure 2:**
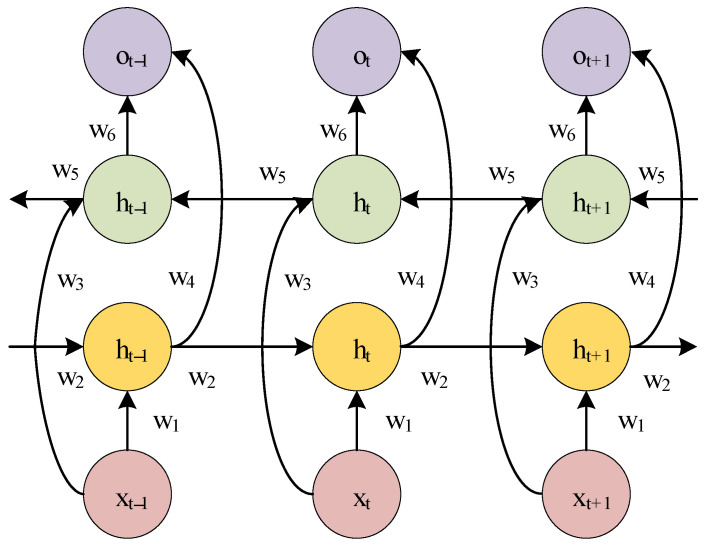
Structure diagram of BiLSTM.

LSTM can solve the gradient problem in RNN, but its structure determines that LSTM can only carry out forward propagation operations. The timing characteristics contained in the vibration signal of the rolling bearing are related to the state of the time before and after. BiLSTM leads the data flow direction from the future to the past on the basis of LSTM. Therefore, BiLSTM can be used to better extract deep information from sequence data. The structure of BiLSTM is shown in Figure 2.

BiLSTM consists of forward LSTM and reverse LSTM. BiLSTM uses the known time series and reverse position series to deepen the feature extraction of the original sequence through the two-way operation of forward and back propagation. The final output of the BiLSTM neural network is the combination of LSTM forward propagation and back propagation. BiLSTM is calculated as follows:

(7) $${\vec{h}}_{t}=f\left(w_{1}x_{t}+w_{2}{\vec{h}}_{t-1}\right)$$

(8) $${\overset{\leftarrow }{h}}_{t}=f\left(w_{3}x_{t}+w_{5}{\overset{\leftarrow }{h}}_{t-1}\right)$$

(9) $$o_{t}=g\left(w_{4}\vec{h_{t}}+w_{6}\overset{\leftarrow }{h_{t}}\right)$$

In the above formula, $w_{x}$ is the weight in BiLSTM, $x_{t}$ is the input at time t, ${\vec{h}}_{t}$ is the forward LSTM hidden layer output at time t, ${\overset{\leftarrow }{h}}_{t}$ is the reverse LSTM hidden layer output at time t, ${\vec{h}}_{t-1}$ is the forward LSTM hidden layer output at time t−1, ${\overset{\leftarrow }{h}}_{t-1}$ is the reverse LSTM hidden layer output at time t−1, and $o_{t}$ is the final output of BiLSTM.

The original data used in this paper are vibration acceleration data, which are time series data. BiLSTM can capture nonlinear features and long-term dependence in time series, reverse LSTM can capture future information in the series, and forward LSTM is responsible for capturing past information. BiLSTM can use both past and future contextual information to process current data points.

### 2.2. Locally Sparse Structure

In the development process of Convolutional Neural Networks, the topology of classical Convolutional Neural Networks is generally set alternately between convolutional layers and pooled layers. A fully connected layer can be set after a certain number of layers are stacked to achieve classification tasks. Convolutional Neural Networks perform convolutional operations through the convolutional kernel and the local region in the convolutional layer and constantly slip to obtain global features. Therefore, increasing the number of convolutional layers and convolutional nuclei in a period of time is a possible method used to improve performance, such as accuracy, but this method will cause parameter redundancy of the network model, resulting in an overfitting phenomenon, and thus reduce the performance of the network. At the same time, it is difficult to perfect the local features extracted from the convolutional nuclei of different scales in a single channel.

**Figure 3:**
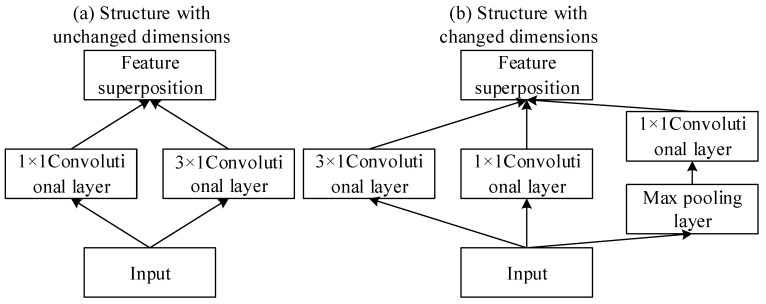
Diagram of local sparse structure with and without dimension change.

According to Hebbian theory [59], the connections between neurons can be achieved through repeated activation between neurons, and neighboring neurons will also be enhanced by this effect; that is, neurons with similar functions tend to cluster together. Therefore, the larger convolutional layer in the Convolutional Neural Network can be transformed into a local sparse structure of multiple neuronal clusters according to Hebbian theory; that is, the larger convolutional kernel can be replaced by a number of smaller convolutional nuclei with similar functions to reduce the parameter redundancy of the model and improve the performance of the model. Two common local sparse structures are shown in Figure 3.

As shown in the figure above, the local sparse structure can be divided into two categories based on the changes in the feature map size before and after input. The output of the convolutional layer is reduced by half in the structure with unchanged dimensions in comparison with the changed dimensions in the structure. The local sparse structure uses small-sized convolution kernels instead of large-sized convolution kernels to represent clusters of neurons with similar functions. By adding convolution kernels of different sizes to the local sparse structure, the different features can be fully extracted in the given information. The convolution layer in the local sparse structure can not only change the output size and reduce parameter redundancy but also realize the feature fusion of different channels. By introducing local sparse structures, the parameters of the Convolutional Neural Network can be reduced, the expressiveness of the network model can be improved, and, eventually, the performance of the model can be improved.

### 2.3. Optimization of Activation Function

In deep learning models, the Rectified Linear Unit (ReLU) activation function and its improved variant are widely used because they can solve the problem of gradient disappearance of network models with faster computation speed in the process of forward and reverse propagation.

**Figure 4:**
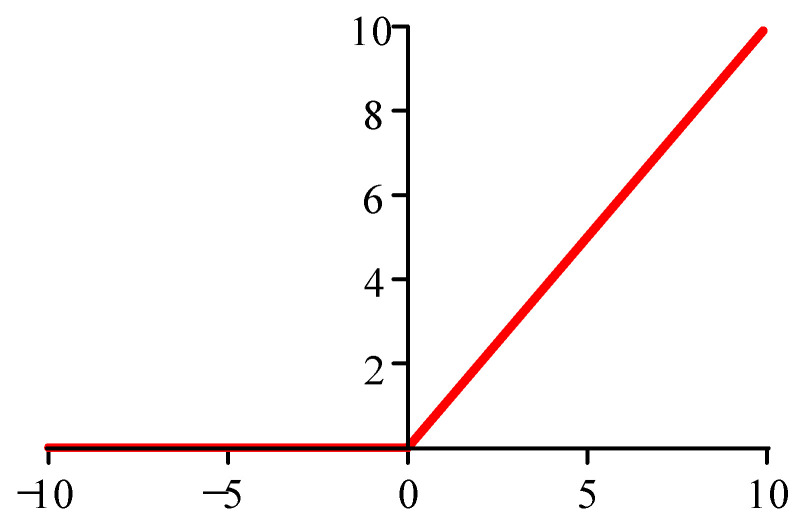
Graph of the ReLU activation function.

However, the value of the gradient of the ReLU function is 0 at x<0, leading to the neuron weight being 0 during the directional propagation of deep learning models. This includes, for example, the phenomenon of neuron necrosis, which may affect the outcome during the training process. The ReLU activation function is shown in Figure 4.

**Figure 5:**
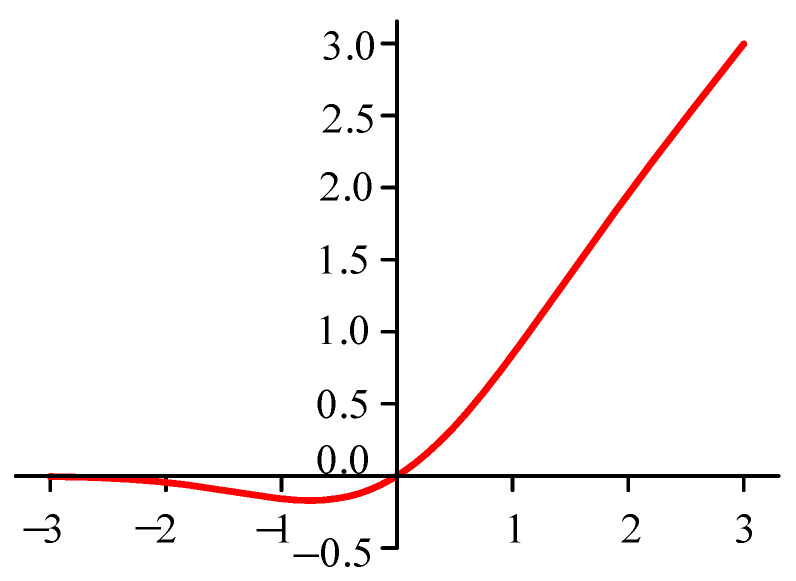
Graph of the GeLU activation function.

The use of the GeLU function can improve the fitting performance of data after normalization. Randomness is increased by introducing the idea of random regularity. Both the GeLU function and the ReLU function pass through the origin, but the GeLU function is smoother and not a piece way function, which reduces mutations at the origin and restrains the influence of the negative activation region. It can be adapted to the large number of negative values in the bearing vibration signal through the Convolutional Neural Network (see Figure 5).

In this section, the GeLU (Gaussian Error Linear Units) function is proposed to replace the traditional ReLU function, and its mathematical expression is as follows:

(10) $$GELU\left(x\right)=x*P\left(X\leq x\right)=x\varphi (x)=x\int_{-\infty }^{x}\frac{\exp\left(-\frac{{\left(X-\mu \right)}^{2}}{2\sigma^{2}}\right)}{\sqrt{2\pi }\sigma }dX$$

In the above formula, 10 is the cumulative distribution function of the Gaussian distribution, 2 is the definite integral of the Gaussian distribution on the interval 3, 4 and 5 are the mean and standard deviation of the normal distribution when the values are 0 and 1, respectively, and the probability function of the standard normal distribution is in the GeLU activation function, whose expression is as follows:

(11) $$GELU\left(x\right)=0.5x\left\{1+\tan h\left[\sqrt{2/\pi }\left(x+0.044715\right)x^{3}\right]\right\}$$

### 2.4. Optimizer Selection

The training process of deep learning algorithms, such as Convolutional Neural Networks, can be quasi-merged with inputs to achieve the optimal solution. In this process, the basic parameters of the model can be constantly updated using the gradient descent method. Common optimizers in deep learning models include Adam, Nesterov-Accelerated Adaptive Moment Estimation, Nadam, Stochastic Gradient Descent (SGD), etc.

The Adam optimizer was proposed by Kingma and Lei Ba in 2014 [60]. The Adam optimizer has the characteristics of adaptive adjustment of the learning rate and good performance when facing most types of tasks. The Nadam optimizer adds the Nesterov momentum term on the basis of the Adam optimizer. The learning rate is adjusted adaptively through second-moment estimation. The Nadam optimizer is conducive to the convergence of the model, and the obtained parameters are stable. The calculation iteration process is as shown in the Algorithm 1.
**Algorithm 1:** Nadam, hyperparameter setting α=0.001, $\mu_{t}=0.9$, v=0.99, $\varepsilon =10^{-8}$Conditions: Initial learning rate α and retention stability constant ε
Conditions: Moment estimate decay rate $\mu_{t}$ and initialization v
Conditions: Initialization of parameter θ
First m=0, Second moment variable n=0
Example Initialize the time step t=0
If $\theta_{t}$ does not converge, perform the following operations:$g_{t}\leftarrow \nabla_{\theta_{t-1}}f_{t}\left(\theta_{t-1}\right)$$m_{t}\leftarrow \mu_{t}\cdot m_{t-1}+\left(1-\mu_{t}\right)g_{t}$$n_{t}\leftarrow v\cdot n_{t-1}+\left(1-v\right){g^{2}}_{t}$${\hat{m}}_{t}\leftarrow \left(\mu_{t+1}m_{t}/\left(1-\overset{t+1}{\underset{i=1}{\prod }}\mu_{i}\right)\right)+\left(\left(1-\mu_{t}\right)g_{t}/1-\overset{t}{\underset{i=1}{\prod }}\mu_{i}\right)$${\hat{n}}_{t}\leftarrow \nu n_{t}/\left(1-\nu^{t}\right)$$\theta_{t}\leftarrow \theta_{t-1}-{\hat{m}}_{t}\cdot \alpha_{t}/\sqrt{{\hat{n}}_{t}+\varepsilon }$End and return $\theta_{t}$


In order to compare the performance of different optimizers, Adam, SGD, and Nadam, three commonly used optimizers, were selected to conduct comparison experiments with the 1DMCICNN model, and the performance of the optimizers was evaluated by comparing the accuracy and loss of the validation sets of different optimizers. The experimental data came from the CWRU dataset, with a speed of 1797 rpm. In total, 24,000 samples were obtained through data enhancement, and 70% and 20% of samples were divided into the training set and the verification set, respectively. The Batch Size was set to 256, and the learning rate was set to 0.001.

**Figure 6:**
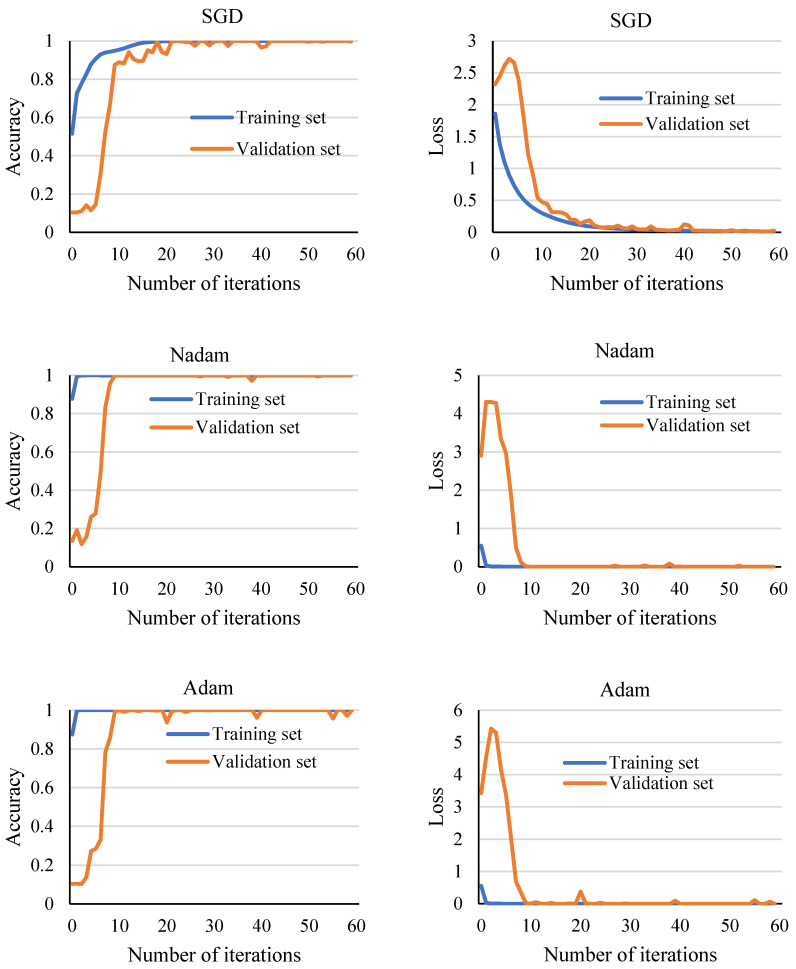
The accuracy of the training set and the verification set corresponding to different learning rates.

As can be seen from the results in Figure 6, when the SGD optimizer is used, the convergence process of the model takes a longer time, and the number of iterations used to achieve convergence is significantly more than that used by the Nadam optimizer and the Adam optimizer. In addition, during the training of the model, the loss value of the verification set has an obvious oscillation phenomenon, and the loss value fluctuation is greater than that used by the other two optimizers. When the Adam optimizer is used, the convergence trend of the model is more obvious, and the number of iterations required is reduced. However, as the number of iterations after model convergence increases, the loss value and accuracy of the validation set fluctuate to a certain extent. When the Nadam optimizer is used, the model converges faster than when the Adam optimizer is used, and convergence is achieved within 10 iterations. The loss value of the verification set is 0.000030, and the loss value of the Adam optimizer is 0.000035. The model performs better in the loss value of the verification set. The model made rapid adjustments and maintained good performance in subsequent training. Therefore, we choose to use the Nadam optimizer in the 1DMCICNN model in this section to obtain better performance.

### 2.5. Sensors Used in the Experimental Platform

**Figure 7:**
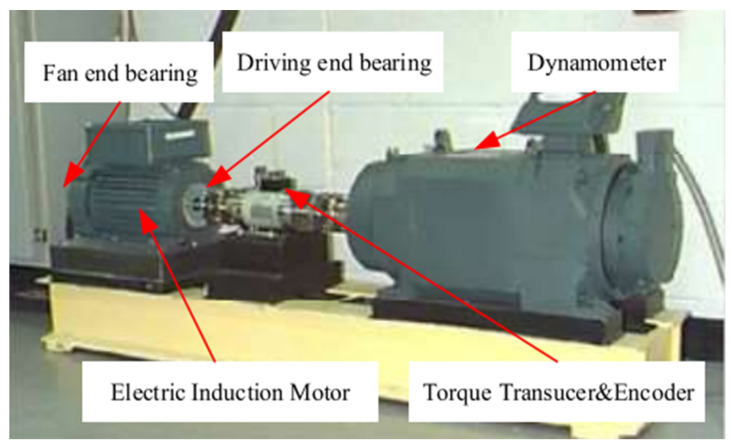
Case Western Reserve University bearing test platform.

The rolling bearing data come from Case Western Reserve University, and the bearing experimental platform is shown in Figure 7. A three-phase induction motor with a power of 1.5 kilowatts is placed on the left side of the test bench. The torque sensor located in the middle of the test bench connects the motors and dynamometers on the left and right sides of the test bench through coupling to achieve torque control. The accelerometer is placed on the motor housing as a sensor, and data are collected through a 16-channel DAT recorder. The bearing models to be tested at the fan end and the drive end are SKF6203 and SKF6205, respectively. In the CWRU rolling bearing dataset, there are three types of faults: the inner ring fault, the outer ring fault, and the rolling element fault. Each fault type includes three fault sizes: 0.007 inches, 0.014 inches, and 0.021 inches. The outer ring fault positions are located at 3:00, 6:00, and 12:00, respectively. For each fault size, four operating conditions are included, namely, 1797 rpm at 0 hp load, 1772 rpm at 1 hp load, 1750 rpm at 2 hp load, and 1730 rpm at 3 hp load.

We collected and recorded the speed and horsepower data using torque sensors and encoders in the CWRU dataset. The torque sensor was mainly used to collect torque information between the drive motor and the load. By analyzing the output signals of the torque sensors, researchers can better understand the vibration signals of rolling bearings under different working conditions, thereby fully verifying the accuracy and anti-interference ability of fault diagnosis.

The accelerometer used in the CWRU dataset is a sensor used to measure and record vibration data of mechanical equipment during operation. The sampling frequency of the accelerometer is 12 kHz and 48 kHz, which means that 12,000 or 48,000 samples were collected per second. The accelerometer records vibration data under different loads and speeds, including bearing operation data under normal conditions and data under various fault conditions.

There are three installation positions for acceleration sensors, namely, on the bearing seat of the motor fan end, the drive end, and the yellow base at the bottom. The data collected at the same time were stored in the same file as the three data channels.

A 16-channel DAT recorder is a device used for data recording and mainly for achieving high-speed, multi-channel, and high-capacity data recording and playback functions. It supports real-time recording and post analysis of data, and it is widely used in various application systems to record various sensor data in industrial production processes for production monitoring and fault diagnosis.

The 16-channel DAT recorder is capable of simultaneously recording data from multiple channels, meeting the requirements for collecting multiple signals. At the same time, due to the high sampling frequency of the gear experimental platform, the 16-channel DAT recorder has a large storage capacity and is suitable for long-term data recording. For the efficiency of subsequent data processing, the recorder supports random playback of data, making it convenient for users to replay and analyze the recorded data.

## 3. Structure and Diagnostic Flow of 1DMCICNN

### 3.1. DMCICNN Structure and Parameter Settings

**Figure 8:**
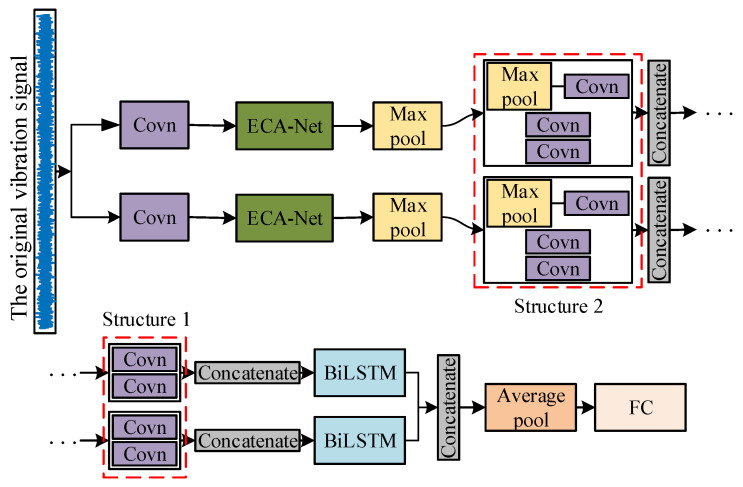
Structure diagram of 1DMCICNN.

In order to extract the features from the original signal more comprehensively and enhance the sensitivity of the model to the time series signal, a new method is proposed as the 1DMCICNN for rolling bearing fault diagnosis. The 1DMCICNN’s structure is shown in Figure 8.

The 1DMCICNN model makes full use of the one-dimensional Convolutional Neural Network’s capacity for good feature extraction for the original bearing vibration signal. Moreover, the feature acquisition capability of the Convolutional Neural Network is further improved by using the convolutional kernel of different scales in the multi-channel network structure, the data loss of the single-channel Convolutional Neural Network is reduced, and the information integrity of the extracted features is improved. This method introduces a local sparse structure in each channel. The model reduces both the probability of an overfitting phenomenon and parameter redundancy. In order to improve the model’s ability to extract fault features from temporal vibration signals, BiLSTM is introduced into the 1DMCICNN. According to the experimental results in this paper, the channel attention mechanism is also introduced into the 1DMCICNN to make the model pay more attention to select key information from datasets.

The 1DMCICNN model contains two parallel channels with different convolution kernel sizes. In each channel, a basic structure of “convolution + pooling” is first set up. To extract features from the original data more comprehensively at different scales, the sizes of the first convolution layers in the two channels are 64 and 128, respectively. After the first convolution layer in each channel, an ECA-Net attention mechanism is added. ECA-Net mainly improves the extrusion and excitation network (SE-Net) module. In SE-Net, two fully connected layers and a nonlinear activation function Sigmoid are used to generate channel weights, and the feature dimension can be reduced to control the complexity of the model. However, this method of dimensionality reduction will cause the loss of some features and increase the calculation amount. ECA-Net uses a 1×1 convolution layer after the global average pooling layer instead of the fully connected layer in SE-Net, which avoids feature dimensionality reduction and captures cross-channel interactions more efficiently. The model can adaptively select the one-dimensional convolution kernel size method, and it can obtain obvious performance gains with only a few parameters added.

Then, two types of local sparse structures with unchanged and changed dimensions are introduced. According to the size of the original data and the size requirements for subsequent processing of the model, the sizes of the convolution kernels and the pooling layers of the two sparse structures are selected through experiments. The parameters of the two local sparse structures in each channel are the same. After the last pooling layer in each channel, the BiLSTM is added to extract the temporal features in the data. The parameters in the BiLSTM are also selected through experiments based on the data format. Then, the information extracted from the two channels is fused and input into the global average pooling layer. Through this method, the global average pooling layer is used instead of the fully connected layer to reduce the training parameters. Finally, the Softmax classifier is used through the fully connected layer to classify the bearing fault types. The specific parameter settings of the network model are shown in Table 1. The local sparse structures with unchanged and changed dimensions are named Structure 1 and Structure 2, respectively.

### 3.2. DMCICNN Fault Diagnosis Flow

**Figure 9:**
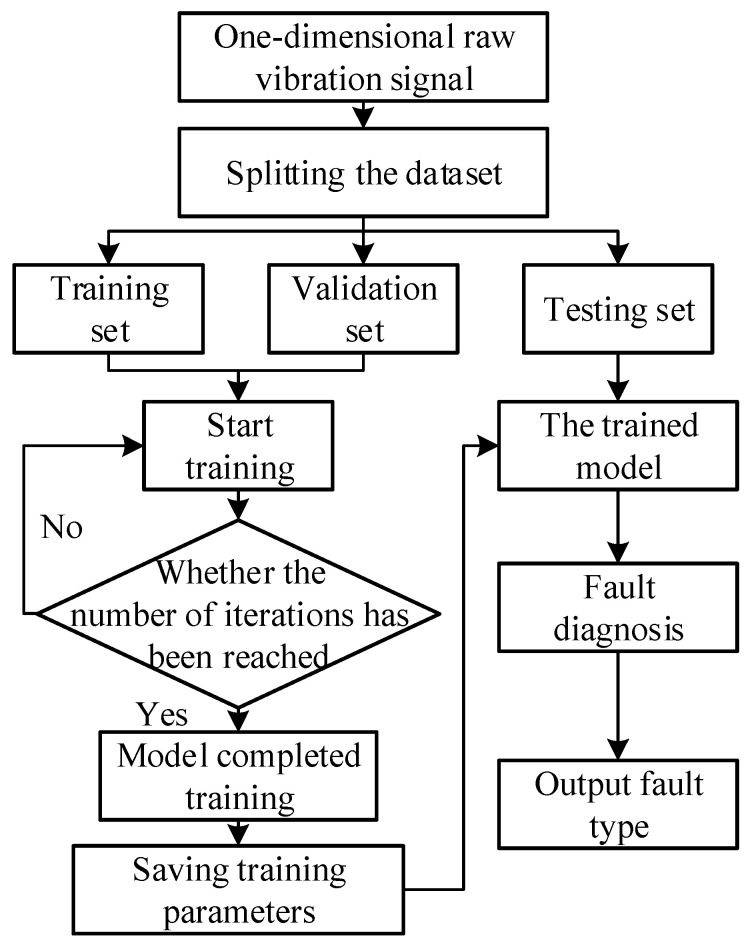
Schematic diagram of the 1DMCICNN fault diagnosis process.

The diagnosis process of the 1DMCICNN model for bearing fault diagnosis is proposed in this section, including two parts: feature extraction and fault type identification. First, the one-dimensional original vibration signal is divided into a training set, a verification set, and a test set. The parameter of noise is added to use the original data for the simulation of a noisy environment in actual working conditions. Then, the deep temporal features in the original datasets are extracted from two optimized parallel multi-channels. After the features are merged, the network parameters are optimized and updated by the average pooling layer and the fully connected layer. The optimal parameters of the model are saved when the predetermined number of iterations is reached. Finally, the test set is input into the trained 1DMCICNN model for fault classification and output. The process framework of the specific experiment is shown in Figure 9.

## 4. Experimental Verification

### 4.1. Summary Description of a Dataset Containing Noise

In the actual working environment of mechanical equipment, noise is unavoidable and random. The network model may learn the noise characteristics from the collected bearing vibration signals, but this results in overfitting, and it may bring challenges to the data-driven deep learning methods that are used for fault diagnosis. In order to reflect the performance of model diagnosis under real working conditions, the experiment is conducted under different intensities of noisy backgrounds with an original vibration dataset including values of noise.

Generally, Additive White Gaussian Noise (AWGN) is added to the model as an original vibration signal to simulate the bearing vibration signal under real working conditions. In AWGN, the average amplitude of power spectral density will obey the Gaussian distribution in the frequency range. In this section, the Signal Noise Ratio (SNR) is used to measure the intensity of noise in the data. The SNR is the ratio of effective signal power to noise power in the data, and its calculation formula is as follows:

(12) $$\mathrm{SNR}=10\log_{10}\left(\frac{P_{\mathrm{signal}}}{P_{\mathrm{noise}}}\right)$$

**Figure 10:**
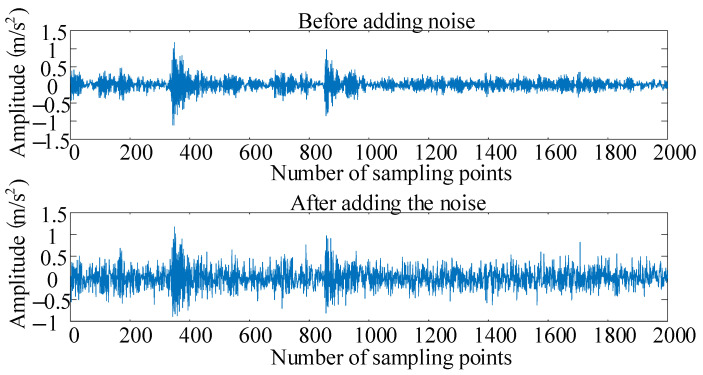
Before and after comparison of Additive White Gaussian Noise in raw data.

Figure 10 shows the signal comparison diagram before and after the addition of AWGN to the original data. The original data are from the bearing dataset of Case Western Reserve University. A sampling frequency of 12 KHz is used to collect signals for the bearing roller at the drive end, and the fault size is 0.021 inch. The speed and the load are 1772 rpm and 1 hp, respectively, and the signal-to-noise ratio is set to −2 dB.

In this section, AWGN is used in the CWRU dataset to simulate noise interference under real conditions. In addition, to illustrate the algorithmic performance in this paper, datasets from the Society for Machinery Failure Prevention Technology (MFPT) will be used. The measured data are the vibration acceleration signals of rolling bearings with the bearing type NICE. The MFPT dataset includes three normal states with a collection time of 6 s, seven outer ring faults with a collection time of 3 s, and seven inner ring fault with a collection time of 3 s. Each fault type is divided into multiple groups of data according to different loads. The working conditions of MFPT data collection are shown in Table 2.

The MFPT dataset and the CWRU bearing dataset were tested using the same noise addition method. In this paper, the CWRU bearing dataset is divided into four datasets, I, J, K, and L, with a sliding window length of 45. Each data sample contains 2048 points, which are the same ten fault types, and each fault type contains 2400 samples. The MFPT dataset used in this paper selects the inner circle fault M dataset under different loads for experiments. In the process of data enhancement, the sliding window length is 50, each data sample contains 2048 points, and each fault type contains 2800 samples. The datasets used in this paper are described in Table 3. In the five datasets, the training set, the verification set, and the test set are divided in a 7:2:1 ratio.

### 4.2. Experimental Process Design

This section contains experiments 1 and 2 using the CWRU and MFPT datasets in Table 3, respectively. According to the experimental comparison results in Section 4.3, the Batch Size was set to 256 and the learning rate was set to 0.001 in the 1DMCICNN model. The Nadam optimizer was selected. The network model was built using the Tensorflow deep learning framework on the Pycharm platform.

In order to illustrate the performance of the 1DMCICNN rolling bearing fault diagnosis method in a noisy environment, in this section, the 1DICNN and the One-Dimensional Multi-Channel Convolutional Neural Network (1DMCCNN), the One-Dimensional Multi-Channel Local Sparse Convolutional Neural Network (1DMCLSCNN), BiLSTM, and the SVM were compared with the methods presented in this section. Local sparse structures are not used in the 1DMCCNN method. In each channel, the two local sparse structures with unchanged dimensions and changed dimensions are replaced by 3×1 convolution cores, after which the maximum pooling layer is formed. The global average pooling layer in front of the classification layer is replaced by a fully connected layer. BiLSTM is not introduced into the 1DMCLSCNN. Other settings are the same as those of the method presented in this section.

### 4.3. Experiment 1 Results and Analysis

In order to compare the fault diagnosis performance of the 1DMCICNN under different signal-to-noise ratios under multiple loads and speeds, additive Gaussian white noise with intensity of −2 dB, 0 dB, 2 dB, 4 dB, 6 dB, and 8 dB is applied to the test set samples of datasets I, J, K, and L. There is no white noise added to the training set or the verification set. The average accuracy of each experiment was obtained ten times to avoid the influence of accidental factors. The experimental results are shown in Table 4.

From the results shown in Table 4, the fault diagnosis accuracy is decreased with the decrease of the SNR for the proposed methods. When the SNR is less than 2 dB, the diagnostic accuracy of the test set has a more obvious declining trend. When the SNR is −2 dB, the average diagnostic accuracy of the four datasets is at least 80.24%. The accuracy of fault identification on different datasets is more than 79%. The average accuracy of the test set reached 99.85% when the SNR was 8 dB. The accuracy of fault identification is stable at more than 98% when the SNR is greater than 4 dB. It is found that the 1DMCICNN has good anti-noise performance according to the model diagnosis results under different signal-to-noise ratios.

**Figure 11:**
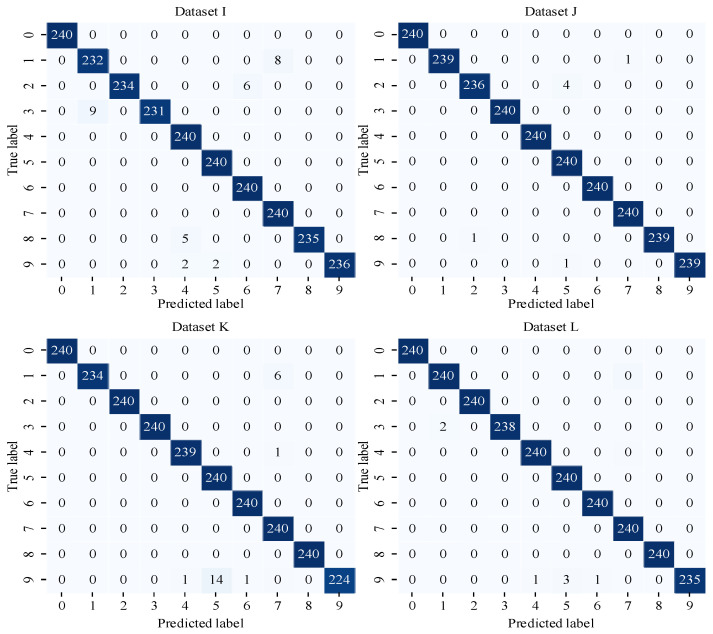
Confusion matrix for model 1DMCICNN when using different datasets.

In order to verify the model’s classification of different fault types, a group of experiments was analyzed using datasets I, J, K, and L with an SNR of 4 dB and a confusion matrix. The results are shown in Figure 11. According to the results in the figure, the proposed model in this paper has achieved an accuracy of 98.67%, 99.71%, 99.04%, and 99.71% in the four datasets with different loads and speeds. In all of the four groups of experiments, the outer ring fault was identified as well as the inner ring fault, and the highest identification error rate was 5.8% when dataset K was used. In dataset L, the overall classification accuracy of the model is as good as 80.15%, 85.20%%, 95.91%, 99.55%, 99.79%, and 99.97%; however, there is still one case of misidentification of an outer ring fault sample. In the four groups of experiments, the model performed well in the identification of all samples under normal conditions because the identification effect of fault samples in the inner circle only has one sample that is wrongly identified. When dataset I was used, the fault samples of the rolling element with different fault sizes were all wrongly assessed, and the lowest identification error rate was 3.2%, which was more accurate for the fault identification of the rolling element in comparison with the other three groups of experiments.

**Figure 12:**
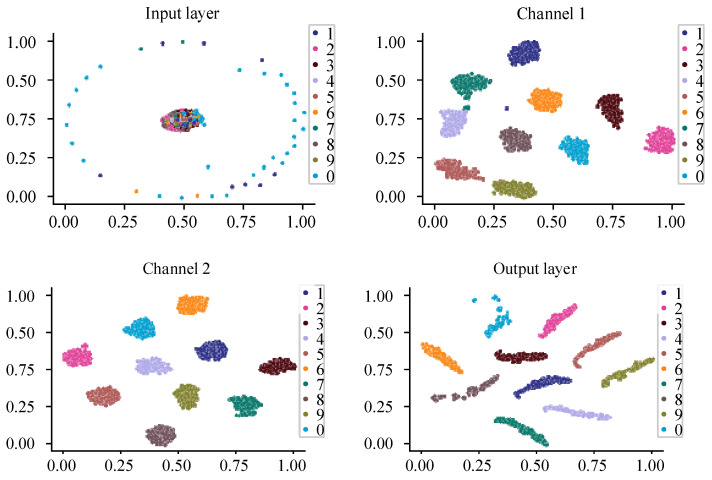
t-SNE feature visualization.

Figure 12 shows the feature distribution of the test data of dataset L in the trained model when SNR is 4 dB. In this section, the t-SNE method is still used to analyze the test set of dataset L through the input layer, the output of channel 1, the output of channel 2, and the output of the fully connected layers.

As can be seen from the results in Figure 9, the variety of different fault types in the test set overlap in a disorderly manner. The sample features extracted through channel 2 have an obvious clustering effect. The feature distribution boundaries of different fault types extracted through channel 1 begin to have clear rules. But, at the same time, some sample feature distributions of the outer ring fault and the inner ring fault still overlap. After the feature fusion of channel 1 and channel 2 through the pooling layer and the output layer, it can be seen that the features of different fault types are further separated and show manifold distribution. The fault data are found to be spatially separable.

**Figure 13:**
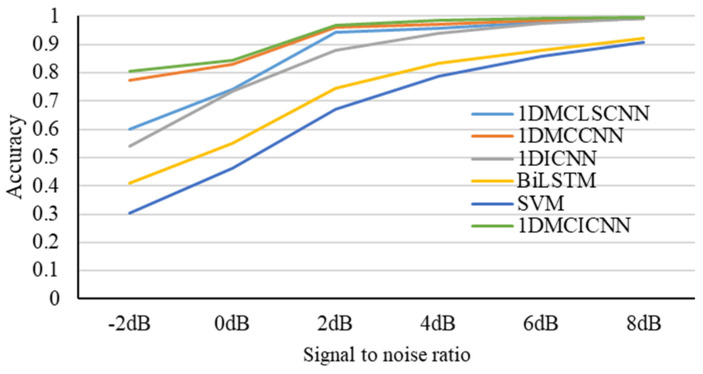
Accuracy curves of the model under different SNR conditions.

In order to further explain the good performance of the method proposed in this paper for the diagnosis of rolling bearing fault, the classical algorithms 1DICNN, 1DMCCNN, 1DMCLSCNN, BiLSTM, and SVM, which are commonly used in bearing fault diagnosis, are compared with the algorithms proposed in this study, and dataset I is selected. Again, only noise is added to the test set. The average accuracy of the test set was obtained 10 times from each group of experiments. The accuracy curve of the model under different SNR conditions is shown in Figure 13. The 1DMCICNN has the best accuracy under different SNR conditions, and it achieves an average accuracy of 80.63% in a high-noise environment with an SNR of −2 dB. The average accuracy was increased to 96.91% when the SNR was 2 dB. The highest accuracy was 99.63% when the SNR was 8 dB. Among the comparison of five different methods, the SVM has the worst performance. Due to strict requirements in parameter selection and kernel function and difficulties in diagnosing various fault types of rolling bearings, the SVM has an average accuracy of only 30.21% in a loud, noisy environment, with a signal-to-noise ratio of −2 dB. The highest average accuracy is 90.64% in a signal-to-noise ratio of 8 dB. But, it is still lower than that of the other five methods. BiLSTM has poor feature extraction ability from original data, and the highest average accuracy is only 92.03%, which is far from the fault diagnosis method based on the CNN. The 1DICNN performs well under high SNR conditions because of its strong feature extraction ability. When the SNR is greater than or equal to 4 dB, the average accuracy is higher than 90%, but the performance is not good under low SNR conditions. There is no BiLSTM in the 1DMCLSCNN to further process the extracted features. Thus, the performance under low SNR conditions is worse than that of the 1DMCCNN. The performance of the 1DMCCNN and the 1DMCICNN is close, with a difference of 3.2% when the SNR is −2 dB. However, due to the local sparse structure and the global average pooling layer, there is a large difference in the number of parameters between the two models in the training process. The pair of parameters is shown in Table 5. In the case of better performance, the number of trainable parameters of the 1DMCICNN decreased by 55.58% compared with the 1DMCCNN. The total number of parameters decreased by 55.83%. It can be seen from the above results that the 1DMCICNN can significantly reduce the number of parameters in the model while maintaining good anti-noise performance in the noisy environment.

**Figure 14:**
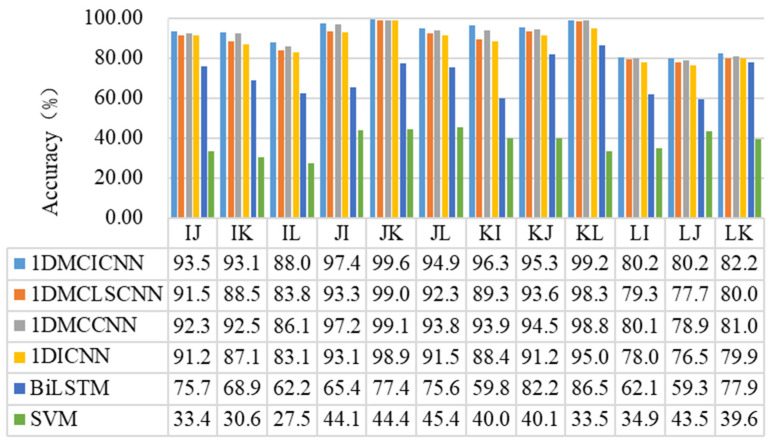
Comparison of accuracy of different models under complex conditions.

In addition to noise interference in the working environment, vibration signals collected by rotating machinery are also affected by factors of various working conditions. In order to study the performance of the method proposed in this section under the combined action of various working conditions and noise factors, the same dataset as above is used in the following experiment. The Additive White Gaussian Noise with an SNR of 4 dB is added to the dataset. One dataset is used for training. One of the other datasets is used as the test set. In order to avoid contingency, average values are taken ten times from each group of experiments. The test set accuracy of the 1DMCICNN and other common methods under the combined conditions of various working conditions and noise is shown in Figure 14.

It can be seen from the results that under the conditions of adding noise and changing working conditions, the 1DMCICNN has achieved the best performance among 12 groups of experiments. The accuracy of eight experimental groups is above 90%, and the accuracy of the remaining four groups is above 80%. The 1DMCICNN uses dataset J as the training set. When dataset K is used as the test set, the highest accuracy is 99.65%, which is better than that of the other three experimental groups when dataset L is used as the training set. When adding noise signals, the difference in the test set features in the two datasets with different loads and speeds changes, resulting in fluctuations in the diagnostic accuracy of the 1DMCICNN model. The 1DMCICNN demonstrated the best diagnostic accuracy in three experiments using dataset L as the training set. For improvements such as use of BiLSTM, attention mechanisms, and multi-channel parallel convolution, the 1DMCCNN also performs better than the 1DMCLSCNN and the 1DICNN within 12 experimental groups. But, the overall performance is worse than that of the 1DMCICNN. When dataset K is used as the training set and dataset I is used as the test set, the accuracy difference between the two models is 2.37%. Based on the comparison of the training parameters above, it can be seen that the 1DMCICNN can achieve better performance under the premise of fewer training parameters under the compound working conditions. BiLSTM and the SVM performed poorly, among which the lowest diagnostic accuracy of the SVM was only 27.59%, and the accuracy of all 12 experimental groups was lower than 46%. BiLSTM was limited by its ability to perform feature extraction, and the performance of the 1DMCLSCNN, 1DMCCNN, and 1DICNN using improved CNN for feature extraction in multiple experiments under complex conditions was significantly better than that of BiLSTM. The above experimental results show that the 1DMCICNN model has better diagnostic performance under complex conditions.

### 4.4. Experiment 2 Results and Analysis

**Figure 15:**
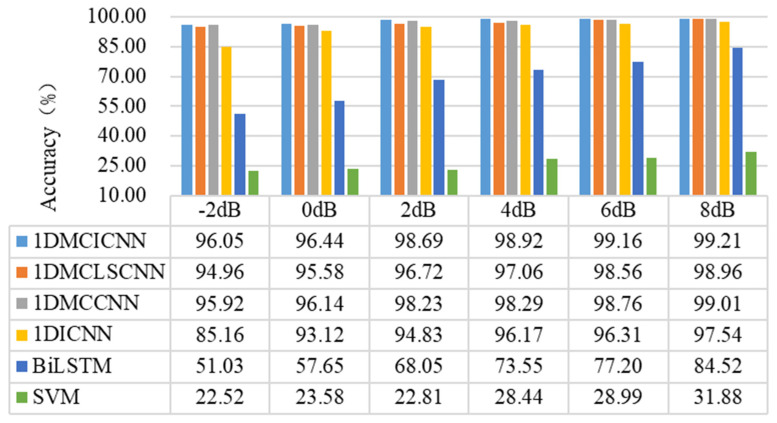
Comparison of the accuracy of different models under different working conditions.

In experiment 2, dataset M from MFPT was used, which contained inner ring fault acceleration data under seven different loads. Six kinds of Additive White Gaussian Noise with different intensities were added to the original signal to test the performance of different models under different working conditions. The experimental results are shown in Figure 15.

As can be seen from the results in Figure 13, the 1DMCICNN has achieved the highest accuracy of fault category identification under the condition of a signal-to-noise ratio of seven different intensities. When SNR is −2 dB, the accuracy can still reach 96.05%, which is 10.89% higher than that of the 1DICNN. It is found that the improved structure has good classification performance for the same type of fault under different working conditions. With the improvement of SNR, the 1DMCICNN reaches 99.21% recognition accuracy when the SNR is 8 dB, which shows a gap between the 1DMCLSCNN and the 1DICNN. This reflects the BiLSTM introduced in the model proposed in this section to improve the performance of feature extraction of time series signals. The performance of the 1DMCICNN is slightly better than that of the 1DMCCNN, indicating that the introduction of a local sparse structure in the model can effectively replace the original convolution kernel, which improves the performance of the model while reducing the parameter redundancy. The SVM and BiLSTM performed poorly, with accuracy lower than 85% under the seven signal-to-noise ratios. Different from other deep learning models, the SVM’s diagnostic accuracy was 22.52% when the signal-to-noise ratio was −2 dB. The recognition accuracy was only 31.88%, even when the signal-to-noise ratio was 8 dB.

**Figure 16:**
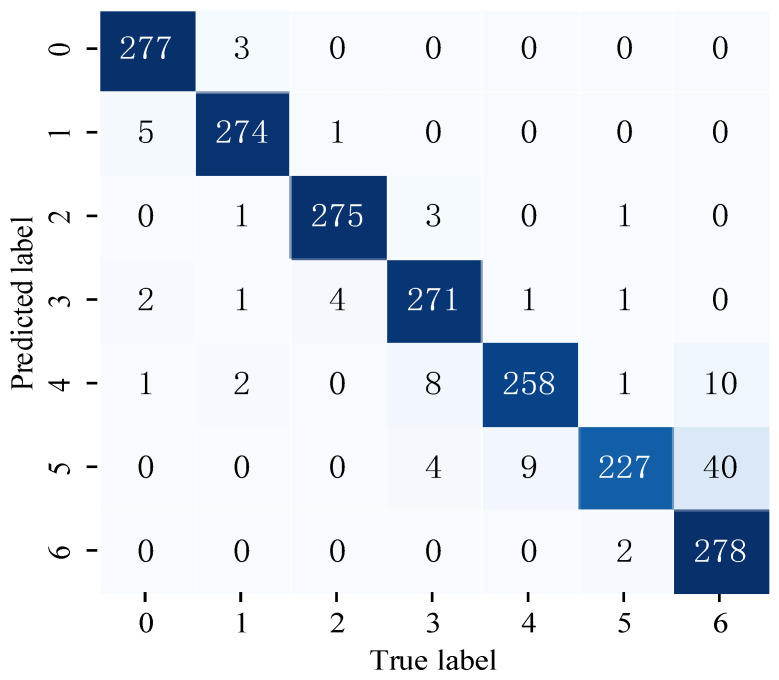
1DMCICNN confusion matrix when using dataset M.

In order to further understand the classification of inner ring fault types under different loads in dataset M using the 1DMCICNN, a group of experiments with an SNR of −2 dB was conducted. The confusion matrix of classification is shown in Figure 16, and the overall accuracy of the model is 95.97%. The 1DMCICNN has the highest classification accuracy of 99.29% for inner ring faults with a 300 lb. load under strong noise. When the load was 250 lb., the accuracy of the model was 81.07%. Because the fault samples with loads of 250 lb. and 300 lb., respectively, had similar characteristics and strong noise interference, the model incorrectly identified many samples as inner ring faults with the load of 300 lb. When the load is 0 lb., 50 lb., 100 lb., and 150 lb., the accuracy of the four fault types exceeds 96.79%.

Based on the above experimental results, it can be proven that the 1DMCICNN has high accuracy and stability for fault identification under various load conditions in noisy environments.

## 5. Evaluation

In this study, we conducted a comprehensive evaluation of the performance of the 1DMCICNN model under various SNR conditions. The experimental results demonstrate that the 1DMCICNN consistently exhibited superior fault category recognition accuracy across seven different SNR scenarios. Specifically, even under the extreme condition of an SNR of −2 dB, the recognition accuracy of the 1DMCICNN remained high at 96.05%, marking a significant improvement of 10.89% compared to the traditional 1DICNN model. This substantial enhancement confirms the 1DMCICNN model’s distinct advantage in classifying the same type of fault features under different working conditions.

As the SNR increased, the performance of the 1DMCICNN was further enhanced. At an SNR of 8 dB, the recognition accuracy of the 1DMCICNN reached 99.21%, showing a clear advantage over the 1DMCLSCNN and the 1DICNN. This outcome is attributed to the incorporation of the Bi-Directional Long Short-Term Memory Network (BiLSTM) in the model proposed in this paper, which excels in the extraction of temporal signal features, thereby significantly enhancing the overall performance of the model.

Furthermore, the performance of the 1DMCICNN slightly outperformed that of the 1DMCCNN, indicating that the introduction of local sparsity structures can effectively replace traditional convolutional kernels. This improvement not only enhances model performance but also reduces parameter redundancy, thereby increasing model efficiency.

In contrast, the Support Vector Machine (SVM) and BiLSTM performed unsatisfactorily under all seven SNR conditions, with accuracy not exceeding 85%. In particular, the SVM showed a diagnostic accuracy of only 22.52% at an SNR of −2 dB, and even at an SNR of 8 dB, the recognition accuracy was a mere 31.88%. This performance gap is significant when compared to deep-learning-based models.

In summary, the 1DMCICNN model demonstrated exceptional fault recognition capabilities under various SNR conditions, with a particularly notable enhancement in performance under low SNR conditions, further attesting to its potential and value in practical applications. These results not only provide a new perspective for the field of fault diagnosis but also lay a solid foundation for future research and applications.

## 6. Conclusions

### 6.1. Conclusion of the Article

In this paper, a fault diagnosis model, the 1DMCICNN, for rolling bearing is proposed, which is suitable for noise interference, various working conditions, and different load conditions. This paper is mainly divided into three parts. The first part introduces the construction and parameter setting of the 1DMCICNN. The 1DMCICNN takes one-dimensional original vibration acceleration data as the input of the model, sets two parallel one-dimensional multi-channels in the model, and introduces the ECA-Net channel attention mechanism after the convolutional layer in the two channels. Two kinds of local sparse structures were used to replace the original convolution kernel in the model, and BiLSTM was introduced into the two channels to further extract the timing information of features. The model is constructed to improve the activation function and optimizer in the model. In the second part, the important hyperparameters of the model are optimized, and the best parameters are selected through the process of experimental comparison. In the third part, the comparison experiments under single working conditions, various working conditions, and different load conditions in noisy environments proved that the proposed method, 1DMCICNN, has good diagnostic accuracy and generalization compared with other methods in noisy environments.

### 6.2. Directions for Future Research

The data used in the fault diagnosis method proposed in this paper can meet the requirements of data sample size after data enhancement so as to achieve better fault diagnosis effects, improve the accuracy of fault diagnosis on the premise of significantly reducing the amount of data, and hardly requiring professional knowledge of users in the fault diagnosis process. However, in actual working conditions, it is often difficult to collect fault samples of mechanical equipment. As a method of supervised learning, it is necessary to label the collected data in advance, which will cause misjudgment of types of faults that have never occurred. In this paper, vibration signals collected by a single vibration sensor in the open dataset are used. Further research is needed on other types of sensor signals used by other types of mechanical equipment.

Based on the above limitations, some scholars have carried out relevant studies, such as Alexandre Martins [61], who proposed to input the collected data into a deep learning model and then use the hidden Markov model to classify the fault modes into early warning, normal, and alarm. This method can detect other abnormal states of the equipment under normal transport conditions. Moreover, this method can be widely used among various types of mechanical equipment and sensors. At the same time, this method uses a variety of machine learning methods, and more data information can be obtained according to the results of data feature conversion in the process of fault prediction and diagnosis. Such methods are more oriented to practical applications, providing a novel angle for subsequent fault diagnosis methods based on deep learning methods.

Although the fault diagnosis method based on deep learning gets rid of the dependence on expert experience compared with the traditional fault diagnosis method, in the actual diagnosis process, the deep learning model still needs to select the best parameters according to the results of several experiments. In order to reduce the time for the deep learning model to confirm the training parameter process, in follow-up research, we can use the meta-heuristic optimization algorithm to automatically optimize the training parameters and further improve the adaptability of the model.

The data-driven deep learning fault diagnosis method proposed in this paper uses vibration acceleration signals used in many studies at present, but there are various other forms of data in rotating machinery that can also be used as data sources for the model. In subsequent research, other forms of data or a combination of multiple forms of data can be used for fault diagnosis research.

The fault diagnosis method based on deep learning proposed in this paper is a black box model. Although relevant studies have proven that it has good performance, the cause and basis of fault classification judgment given by the model are difficult to explain. In the subsequent work, the interpretability of deep learning can be improved from different perspectives to promote the practical application of deep learning methods in the field of fault diagnosis.

## Figures and Tables

**Table 1:** Parameters of 1DMCICNN.

Name		Kernel Size	Step Size	Input Size	Output Size	Number of Convolution Kernels
Input 1		-	-	2048 × 1	2048 × 1	-
Input 2		-	-	2048 × 1	2048 × 1	-
Covn 1-1		64	16	2048 × 1	128 × 32	32
ECA-Net		-	-	128 × 32	128 × 32	-
Max pool 1-2		2	2	128 × 32	64 × 32	-
Covn 2-1		128	16	2048 × 1	128 × 32	32
ECA-Net		-	-	128 × 32	128 × 32	
Max pool 2-2		2	2	128 × 32	64 × 32	-
$\mathrm{Structure}\;2\{$	Max pool	2	1	64 × 32	64 × 32	-
	Covn	1	2	64 × 32	32 × 32	32
	Covn	3	2	64 × 32	32 × 16	16
	Covn	1	2	64 × 32	32 × 64	64
Concatenate 1		-	-	-	32 × 112	-
$\mathrm{Structure}\;1\{$	Covn	3	1	32 × 112	16 × 64	64
	Covn	1	1	32 × 112	16 × 32	32
Concatenate 2		-	-	-	16 × 96	-
BiLSTM 1-5				16 × 96	16 × 32	-
$\mathrm{Structure}\;2\{$	Max pool	2	1	64 × 32	64 × 32	-
	Covn	1	2	64 × 32	32 × 32	32
	Covn	3	2	64 × 32	32 × 32	32
	Covn	1	2	64 × 32	32 × 64	64
Concatenate 3		-	-	-	32 × 128	-
$\mathrm{Structure}\;1\{$	Covn	3	1	32 × 128	16 × 64	64
	Covn	1	1	32 × 128	16 × 32	32
Concatenate 4		-	-	-	16 × 96	-
BiLSTM 2-5		-	-	16 × 96	16 × 32	-
Concatenate 7		-	-	-	16 × 64	-
Average pool		-	-	16 × 64	64	-
FC		-	-	64	10	-

**Table 2:** MFPT dataset at the time of collection.

Type of Fault	Load (lb)	Rotational Speed (Hz)	Sampling Frequency (Hz)
Normal	270	25	97,656
Inner loop	0/50/10/1150/200/250/300	25	48,848
Outer ring	25/50/10/1150/200/250/300	25	48,828

**Table 3:** Description of the datasets used in experiments 1 and 2.

Data Sources	Bearing Type	Dataset	Fault Size (Inch)/Load (lb)	Fault Type	Label	Sample Size
CWRU	SKF6205	I	-	Normal	0	2400
			0.007/0.014/0.021	Rolling element	1/2/3	7200
			0.007/0.014/0.021	Inner ring	4/5/6	7200
			0.007/0.014/0.021	Outer ring	7/8/9	7200
		J	-	Normal	0	2400
			0.007/0.014/0.021	Rolling element	1/2/3	7200
			0.007/0.014/0.021	Inner ring	4/5/6	7200
			0.007/0.014/0.021	Outer ring	7/8/9	7200
		K	-	Normal	0	2400
			0.007/0.014/0.021	Rolling element	1/2/3	7200
			0.007/0.014/0.021	Inner ring	4/5/6	7200
			0.007/0.014/0.021	Outer ring	7/8/9	7200
		L	-	Normal	0	2400
			0.007/0.014/0.021	Rolling element	1/2/3	7200
			0.007/0.014/0.021	Inner ring	4/5/6	7200
			0.007/0.014/0.021	Outer ring	7/8/9	7200
MFPT	NICE	M	0/50/100/150/ 200/250/30	Inner ring	0/1/2/3/4/5/6	19,600

**Table 4:** Fault diagnosis results under different SNR conditions.

Dataset	Accuracy (%)					
	−2 dB	0 dB	2 dB	4 dB	6 dB	8 dB
I	80.63	84.39	96.91	98.66	99.29	99.63
J	81.08	83.10	95.60	99.47	99.60	99.93
K	79.08	84.90	95.44	99.10	99.83	99.87
L	80.15	85.20	95.91	99.55	99.79	99.97
Average value	80.24	84.40	95.97	99.20	99.63	99.85

**Table 5:** Comparison of parameters of different models.

Number of Parameters	1DMCCNN	1DMCICNN
Total number of parameters	231,248	102,720
Number of trainable parameters	230,352	101,728
Number of non-trainable parameters	896	992

## Data Availability

All data generated or analyzed during this study are included in this published article and its supplementary information files. If there are other data requirements, answers can be obtained from the corresponding author. The data from CWRU can be obtained at https://engineering.case.edu/bearingdatacenter/download-data-file (accessed on 1 November 2024).
